# Environmental Benefits of Yeast Postbiotic *Saccharomyces cerevisiae* in Broiler Production: A Life Cycle Assessment Approach

**DOI:** 10.3390/ani16152360

**Published:** 2026-08-02

**Authors:** J. Alberto Conde-Aguilera, Céline Garat Boute, Alain Riggi, Charline Dazeur, Agnes Mori

**Affiliations:** Phileo by Lesaffre, 59700 Marcq-en-Baroeul, France; c.garatboute@phileo.lesaffre.com (C.G.B.); a.riggi@phileo.lesaffre.com (A.R.); c.dazeur@phileo.lesaffre.com (C.D.); a.mori@phileo.lesaffre.com (A.M.)

**Keywords:** *Saccharomyces cerevisiae*, postbiotics, life cycle assessment, broiler chicken, environmental impact, *Clostridium perfringens*, heat stress

## Abstract

With increasing meat demand and global warming, the sustainability of broiler chicken production has become a “hot topic” for the conventional industry. We conducted a life cycle assessment (LCA) to compare the environmental impact of supplementing broiler chicken diets with postbiotics derived from the yeast cell wall (YCW). Supplementation with *S. cerevisiae* reduced the carbon footprint of climate change (8.4%), land use (8.7%), water use (7.7%), resource use (7.9%), acidification (12.5%), marine eutrophication (9.5%), freshwater eutrophication (8.6%), terrestrial eutrophication (12.6%), and particulate matter (11.4%). The positive effects on the environmental impact were associated with improvement in the feed conversion ratio (FCR: −7.6%), body weight gain (BWG: +5.7%) and a reduction in mortality of around 44.1%. Overall, the environmental benefit was similar in chickens exposed to heat stress (−8.7%) or challenged with *Clostridium perfringens* (−8.2%), two stressful situations commonly encountered on poultry farms. This LCA study demonstrates that *S. cerevisiae* is a food additive that can help the poultry industry cope with stress caused by various factors and reduce its overall environmental impact.

## Simple Summary

Environmental sustainability is one of the most important concerns worldwide that requires special attention in all of the economic sectors. In this context, postbiotics have gained interest as an alternative to growth-promoting antibiotics due to their ability to enhance nutrient utilization and inhibit pathogen proliferation and their antioxidant properties. Optimizing growth performance, such as enhancing the feed conversion ratio, lowers climate impacts. The main goal of this study was to compare the environmental impact of postbiotic supplementation in the diet of broiler chickens, using a specific strain of yeast, *Saccharomyces cerevisiae*. Life cycle assessment, which is a method to evaluate the environmental impact of products, was used in this study from the extraction of raw materials to the point when the chicken leaves the farm (cradle-to-farm gate). Six studies conducted in different countries and assessing two conditions, heat stress and *Clostridium perfringens* challenge, were analyzed. The results showed that the supplementation of *S. cerevisiae* to the diet reduced the overall carbon footprint by 8.4%, corresponding to 0.19 kg CO_2_ eq per kg of live weight broiler, and was observed in all categories. Meanwhile, *S. cerevisiae* improved growth performance and reduced mortality under all conditions. This work provides important data that can improve broiler production systems regardless of the conditions and the geographical location.

## 1. Introduction

To meet the growing demand for animal protein, broiler chicken production has become the cornerstone of the global poultry industry [1,2]. However, it faces interconnected challenges that threaten productivity, animal welfare, and environmental sustainability [3,4]. Among these challenges, efficient growth management is central to ensuring optimal feed conversion ratios (FCRs) and maintaining profitability. Despite advances in production efficiency, environmental impact remains a pressing concern in broiler production. Large-scale production contributes to greenhouse gas emissions, water pollution from nutrient runoff, and extensive land use, raising critical questions about the sector’s long-term sustainability [5].

Heat stress, driven by global warming and particularly prevalent in regions with elevated temperatures, exacerbates these challenges [6,7]. Heat stress impairs feed digestion and immune system functioning in poultry [8]. As broilers are highly sensitive to thermal conditions, heat stress can lead to reduced feed intake, body weight gain (BWG), increased mortality rates, and compromised product quality [9,10]. Thermal stress also excessively increases the formation of reactive oxygen species, which are associated with inflammatory reactions [11], causing dysbacteriosis and increasing both the severity and incidence of necrotic enteritis (NE) lesions [12], responsible for high antibiotic use. The European Union has banned the application of antibiotics in livestock farming since their excessive use led to changes in intestinal flora, the development of antibiotic-resistant bacteria, and high residue levels in animal products [13].

To address these environmental challenges and optimize growth performance with a cost-effective approach, postbiotics have become essential in sustainable poultry farming [14]. Postbiotics, which are non-viable microbial cells or cell components, offer several benefits, such as serving as substitutes for antibiotics, providing extended shelf life, and ensuring high-temperature stability [15]. *S. cerevisiae*, a species of yeast, and its derivatives have received increased attention as postbiotics in poultry farming. Studies have shown that these bioactive derivatives can improve gut microbiota composition through their bacteriostatic and bactericidal activities [16]. Amongst these components, mannan oligosaccharides (MOSs) can bind to bacterial receptors, preventing intestinal colonization by harmful bacteria such as *E. coli* and *Salmonella* [17]. By improving gut integrity and modulating the intestinal immune response, they reduce the risk of NE caused by *Cl. perfringens* [18]. In addition, beta-glucans, a major component of YCW, stimulate the immune system by activating a wide range of immune cells [19]. Alongside their protective function, MOSs and beta-glucan supplementation have resulted in better FCR and BWG, partly due to improved digestibility through enhanced intestinal function and nutrient utilization [20]. Moreover, postbiotics possess antioxidant properties, which help reduce oxidative stress and support birds’ physiological resilience in high-temperature environments [21]. A specific *S. cerevisiae* derivative (produced by Phileo, Lesaffre) is a YCW fraction, rich in MOSs and beta-glucans (1,3- and 1,6-linked), obtained from the primary culture and purification of a selected proprietary strain of *S. cerevisiae*. Previous studies have investigated the effects of *S. cerevisiae* on the production and performance of broiler chickens and laying hens [22,23,24,25].

The use of postbiotics to improve health and promote growth has been well investigated [26,27], but there are few publications assessing their direct impact on carbon footprint. While recent studies have highlighted the potential of natural plant extracts and novel feed ingredients in enhancing gut health and contributing to sustainable livestock production [28,29], a comprehensive life cycle assessment (LCA) is required to quantify the systemic environmental benefits of this specific yeast postbiotic in poultry. Notably, previous LCA studies have already demonstrated the environmental benefits of this specific yeast probiotic (*S. cerevisiae*) in ruminant production, showing significant reductions in carbon footprint and resource use in both dairy cows [30] and beef cattle [31]. However, its direct impact on the environmental footprint of broiler chicken production under stress conditions remains unexplored. We hypothesized that supplementing broiler diets with *S. cerevisiae* postbiotics would improve zootechnical performance metrics, thereby reducing the overall environmental footprint per kg of live weight gain compared to unsupplemented controls. Therefore, this study aimed to apply an LCA approach to evaluate the environmental impacts and growth performance of broiler chickens with or without *S. cerevisiae* supplementation.

## 2. Materials and Methods

### 2.1. System Description

**Figure 1:**
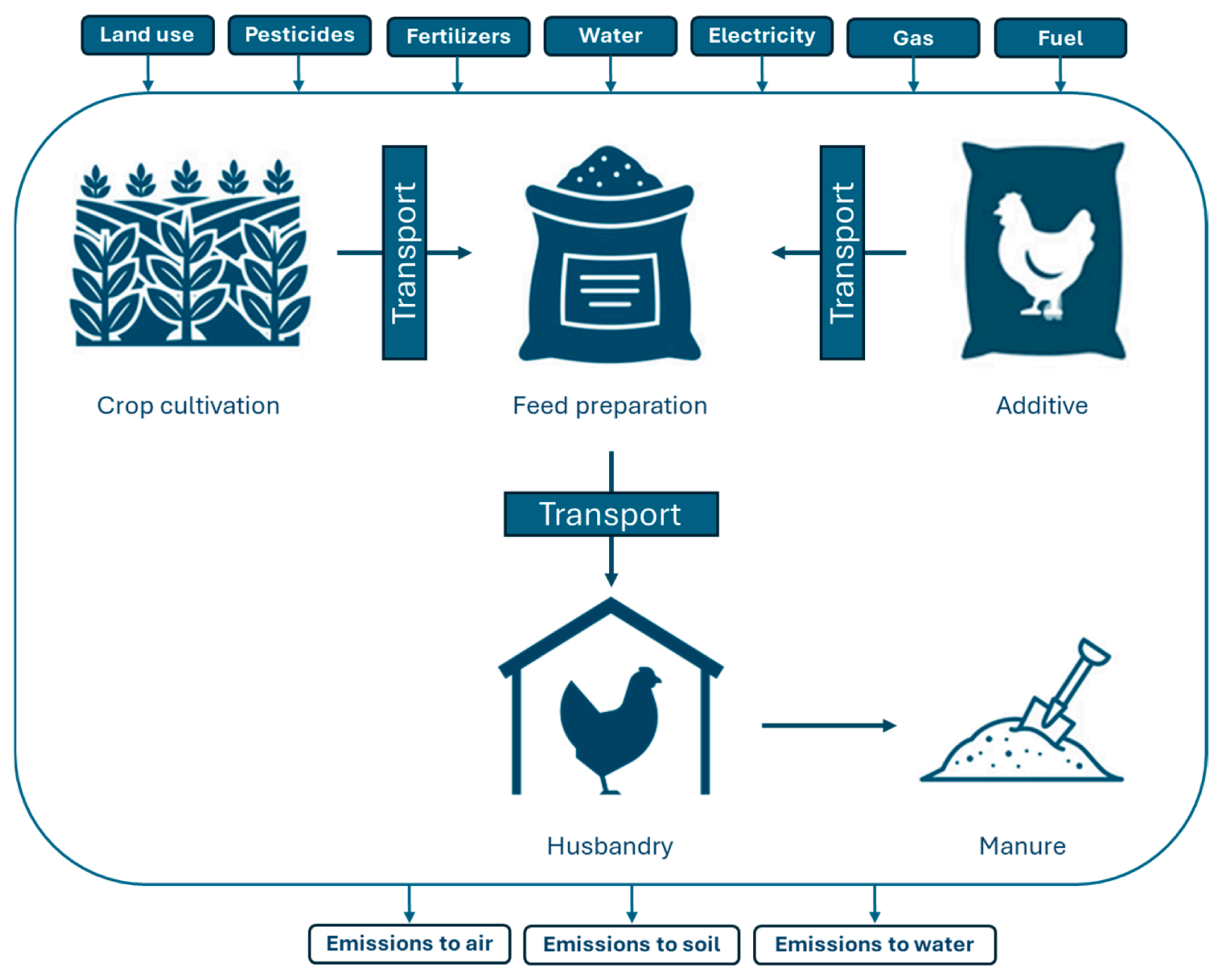
System boundaries of broiler chicken production. Each production system is divided into 5 processes: crop cultivation, feed preparation, production of additive (yeast cell wall fraction), animal husbandry and manure management.

The livestock husbandry systems analyzed represent typical large-scale production systems in three regions of the world: North America, South America and Asia. A simplified overview of the system boundary, represented in Figure 1, was defined as “cradle-to-farm gate”, meaning that all material processes from cultivation (including upstream inputs) to the farm gate were considered. The analysis included all “upstream” activities from crop cultivation to the storage of *S. cerevisiae* at the factory (including transportation) and “downstream” activities such as farm-level operations, including feeding, rearing, and harvesting of broilers. Further “downstream” activities such as processing, distribution, or consumption of animals were not taken into account, as the definition stops at the farm gate. Upstream and downstream data were sourced from Agri-Footprint v6 [32] and Ecoinvent 3.8. Capital goods, such as buildings and machinery, were excluded from the assessment, as recommended by the Product Environmental Footprint Category Rules (PEFCR) guidelines [33].

### 2.2. Life Cycle Assessment General Principles

Life cycle assessment (LCA) is a method used to evaluate the environmental impact across the entire life cycle of a product. The principles and guidelines are established in the international standards (ISO 14040 [34] and ISO 14044 [35]) There are four phases in an LCA study: (1) Goal and Scope Definition, including the specification of functional units and system boundaries, (2) Life Cycle Inventory (LCI), which involve gathering input and output data for all relevant processes; (3) Life Cycle Impact Assessment (LCIA), which quantifies the environmental impacts; and (4) Interpretation, which includes analyzing and validating the results (Figure 1).

### 2.3. Goal and Scope Definition

The goal of the present study was to evaluate the environmental impact of broiler chicken production under two conditions (heat stress or challenge with *Cl. perfringens*), with or without *S. cerevisiae* supplementation. The functional unit was defined as 1 kg of live weight gain (LWG) in broilers at the farm level. Economic allocation was applied to the stages of feed cultivation (main crop and crop residue) and feed processing (grain and hulls), in line with the PEFCRs for animal feed. At the farm level, allocation was based on system separation to attribute environmental impacts between broiler meat and other co-products, such as manure. The assessment period was 42 days (corresponding to one production cycle, with seven cycles per year, excluding the service time).

We assumed that feed transportation from the feed mill to the farm was an average distance of 80 km. Due to the absence of primary data, the gross energy content of the feed was based on the IPCC average value of 18.45 MJ/kg feed. Because the production of *S. cerevisiae* was not initially part of the present study, a proxy value estimated to 1.3 kg CO_2_ eq/kg (based on ex factory conditions) was applied to represent the additive. According to an internal study, utilities used at the farm, including water, electricity, gas, and bedding material consumption, were based on secondary data. However, these inputs are characterized by region-specific secondary processes that depend on the location of the trials.

### 2.4. Life Cycle Inventory

The inventory data for compound feed processing consisted of agricultural datasets sourced from Agri-Footprint v6 for each individual ingredient in the feed formulations. These datasets encompassed inputs and resources (such as crop yield, water utilization, land occupation and transformation, application of manure, fertilizers, lime, pesticides, starter materials, energy, and transport of inputs). Emissions arising from these inputs—including nitrous oxide (N_2_O_x_), ammonia (NH_3_), nitrate (N0_3_^−^), nitric oxide (NO_x_), carbon dioxide (CO_2_), phosphorus (P), pesticide residues, and heavy metals—were also included. Furthermore, emissions resulting from land-use changes and peat oxidation were taken into account.

#### 2.4.1. Feed Production and Compound Feed Processing

Energy requirements for compound feed processing, including electricity and heat, were modeled based on average industrial consumption data from the Agri-Footprint 6 database. While thermal processing can induce chemical reactions such as the Maillard reaction [36], these effects were not explicitly modeled in the present LCA. Specifically, the following values were applied:

- Electricity consumption: 0.041 kWh per kg of compound feed produced.
- Thermal energy (heat): 0.99 MJ per kg of compound feed produced.

These values represent industry averages for modern feed-milling operations, including grinding, mixing, pelleting, and cooling processes. Country-specific energy mix data were applied based on the geographical location of each trial.

#### 2.4.2. Regional Reference Systems from Agri-Footprint v6

Regional reference systems are standardized life cycle inventory datasets embedded in the Agri-Footprint v6 database that represent average production systems for specific geographical regions and agricultural commodities. These reference systems include the following:

- Average feed formulations for specific animal categories and production stages.
- Typical performance parameters (growth rates, feed conversion ratios, and mortality rates).
- Standard management practices (housing systems, manure management, and energy use).
- Regional-specific input data (energy mix, transportation distances, and input application rates).
- Average emission factors based on regional conditions.

For this study, the regional reference systems were used to (1) supplement trial data for parameters not measured in the trials; (2) provide background data for one-day-old chick production; (3) model utilities consumption (water, electricity, and gas) based on regional averages; and (4) complete the life cycle inventory for scenarios where primary data were not available. The reference systems ensure geographical consistency and represent typical production conditions in the United States, Mexico, Brazil and Pakistan, corresponding to the locations of the six trials included in this study.

#### 2.4.3. On-Farm Emissions Modeling

The trials lasted from chick placement to slaughter (6 weeks), and emissions related to enteric fermentation, manure management, and energy use were calculated according to Intergovernmental Panel on Climate Change (IPCC) [37] and European Monitoring and Evaluation Program/European Environment Agency (EMEP/EEA) guidelines [38]. Although the IPCC Sixth Assessment Report [39] provides updated values, the conservative values from the 2006 IPCC Guidelines were retained in this study to ensure comparability with prior poultry LCA studies that followed the same methodological framework. These captured emissions included methane (CH_4_), direct nitrous oxide (N_2_O), nitrogen oxides (NO_x_), non-methane volatile organic compounds (NMVOCs), and particulate matter (TSP, PM_2.5_, and PM_10_) from manure management systems as well as poultry housing. Additionally, indirect N_2_O emissions resulting from volatilization of NH_3_ and NO_x_ were incorporated into this analysis.

Utilities consumed on-farm (water, electricity, gas, and bedding material) were modeled using country-specific background processes from Agri-Footprint v6 and Ecoinvent 3.8 databases. The specific consumption rates were as follows:

- Water: 0.0037 m^3^ per kg broiler liveweight output.
- Electricity: 0.041 kWh per kg broiler liveweight output.
- Natural gas: 0.99 MJ per kg broiler liveweight output.
- Bedding material (straw): 0.079 kg per kg broiler liveweight output.

#### 2.4.4. Yeast Postbiotic Production Inventory

The production of the yeast postbiotic additive (Safmannan^®^, Phileo by Lesaffre, Marcq-en-Barœul, France) was modeled using a proxy carbon footprint value of 1.3 kg CO_2_ eq per kg of additive. This value was derived from industry-specific life cycle inventory data for similar fermentation-based feed additives [40]. The estimation encompasses upstream processes (raw material cultivation and transport), production processes (fermentation, centrifugation, and drying), and infrastructure utilities (electricity, thermal energy, and water treatment). Specifically, the inventory includes electricity consumption (estimated at 2.5 kWh per kg of product) and thermal energy for drying (estimated at 15 MJ per kg of product). A sensitivity analysis was performed to test the influence of this assumption on the overall results (see Section 2.8), confirming that variations in this proxy value do not alter the study conclusions.

### 2.5. Calculation Methods and Equations

The following equations were applied to calculate key parameters in this LCA study.

Feed conversion ratio (*FCR*):

(1) $$FCR=\frac{\mathrm{Total}\;\mathrm{Feed}\;\mathrm{Intake}\;(\mathrm{kg}\;\mathrm{DM})}{\mathrm{Total}\;\mathrm{Body}\;\mathrm{Weight}\;\mathrm{Gain}\;\left(\mathrm{kg}\right)}$$

Feed intake is expressed on a dry matter (DM basis), and body weight gain is calculated as the difference between final and initial body weights, adjusted for mortality.

Carbon footprint per kg liveweight:

(2) $$CF=\frac{\sum ({\mathrm{Emission}}_{i}\times {\mathrm{GWP}}_{i})}{\mathrm{Total}\;\mathrm{Liveweight}\;\mathrm{Output}}$$

where carbon footprint (*CF*; kg CO_2_ eq per kg liveweight); Emission*_i_* = quantity of greenhouse gas i emitted (kg); and GWP*_i_* = global warming potential of gas over 100 years (CO_2_ = 1, CH_4_ = 34, and N_2_O = 298) [37].

Methane emissions from manure (IPCC Tier 2):

(3) $$CH_{4}=\sum (N_{\mathrm{animal}}\times VS\times B_{0}\times 0.67\times MCF)$$

where *N*_animal_ = number of animals, *VS*. = volatile solids excreted per animal per day (kg VS/animal/day), *B*_0_ = maximum methane-producing capacity (0.24 m^3^ CH_4_/kg vs. for poultry), 0.67 = conversion factor from m^3^ CH_4_ to kg CH_4_, and *MCF* = methane conversion factor for the manure management system.

Nitrous oxide emissions (direct):

(4) $$N_{2}O_{\mathrm{direct}}=N_{\mathrm{excreted}}\times EF\times \frac{44}{28}$$

where *N*_excreted_ = total nitrogen excreted (kg N), *EF* = emission factor (0.001 kg N_2_O-N per kg N for poultry manure), and 44/28 = conversion factor from N_2_O−N to N_2_O.

Nitrous oxide emissions (indirect from volatilization):

(5) $$N_{2}O_{\mathrm{indirect}}=N_{\mathrm{excreted}}\times {\mathrm{Frac}}_{\mathrm{volatilized}}\times EF_{\mathrm{volatilization}}\times \frac{44}{28}$$

where Frac_volatilized_ = fraction of nitrogen that volatilized as NH_3_ and NO_x_ (0.4 for poultry, according to IPCC 2019 Refinement), and EF_volatilized_ = emission factor for volatilized nitrogen (0.01 kg N_2_O−N per kg N).

Land-use change emissions:

(6) $$LUC_{\mathrm{emissions}}=\frac{\sum ({\mathrm{Area}}_{\mathrm{converted}}\times {\mathrm{Stock}}_{\mathrm{change}}\times \frac{44}{12})}{\mathrm{Annual}\;\mathrm{Production}}$$

where Area_converted_ = area of land converted (ha), Stock_change_ = change in carbon stock (tons C per ha), 44/12 = conversion factor from C to CO_2_, and Annual Production = annual production on converted land (tons).

Allocation for co-products (economic):

(7) $${\mathrm{Impact}}_{\mathrm{product}}=\mathrm{Total}\;\mathrm{Impact}\times \left(\frac{{\mathrm{Value}}_{\mathrm{product}}}{\sum {\mathrm{Value}}_{\mathrm{all}\_\mathrm{products}}}\right)$$

where Value = economic value (price × quantity) of each co-product.

Uncertainty propagation: For the uncertainty analysis, the combined standard uncertainty was calculated as

(8) $$u_{c}=\sqrt{\sum {\left(\frac{\partial f}{\partial x_{i}}\right)}^{2}\times u(x_{i}{)}^{2}}$$

where *u_c_* = combined standard uncertainty, $\partial f/\partial x_{i}$= partial derivative of the function with respect to input *i*, and *u_i_* = standard uncertainty of input *i*.

These equations were implemented in the life cycle assessment software using the Environmental Footprint 3.1 adapted method, with all calculations performed according to ISO 14040 [34] and 14044 standards [35].

### 2.6. Life Cycle Impact Assessment

**Figure 2:**
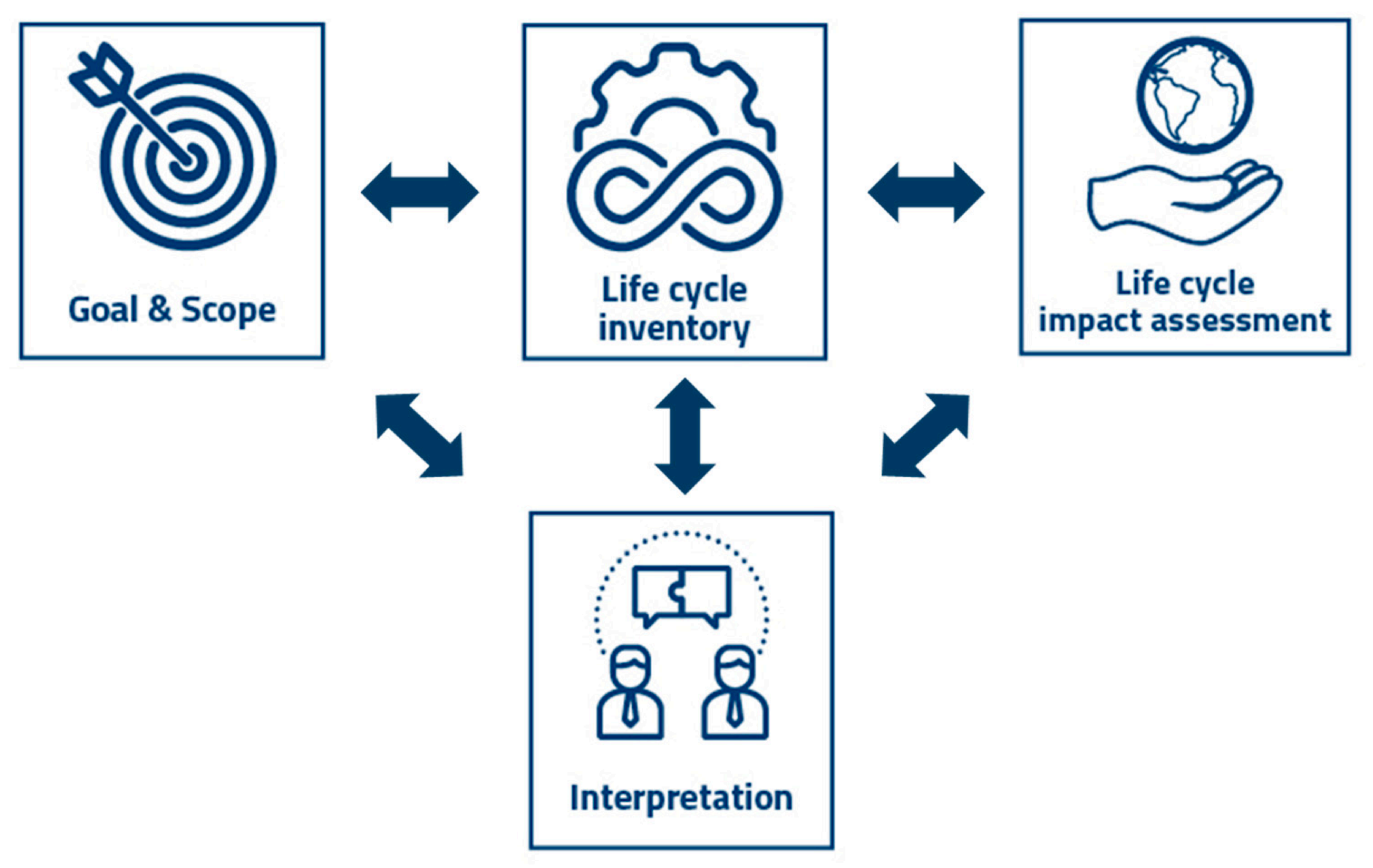
Life cycle assessment process diagram.

**Figure 3:**
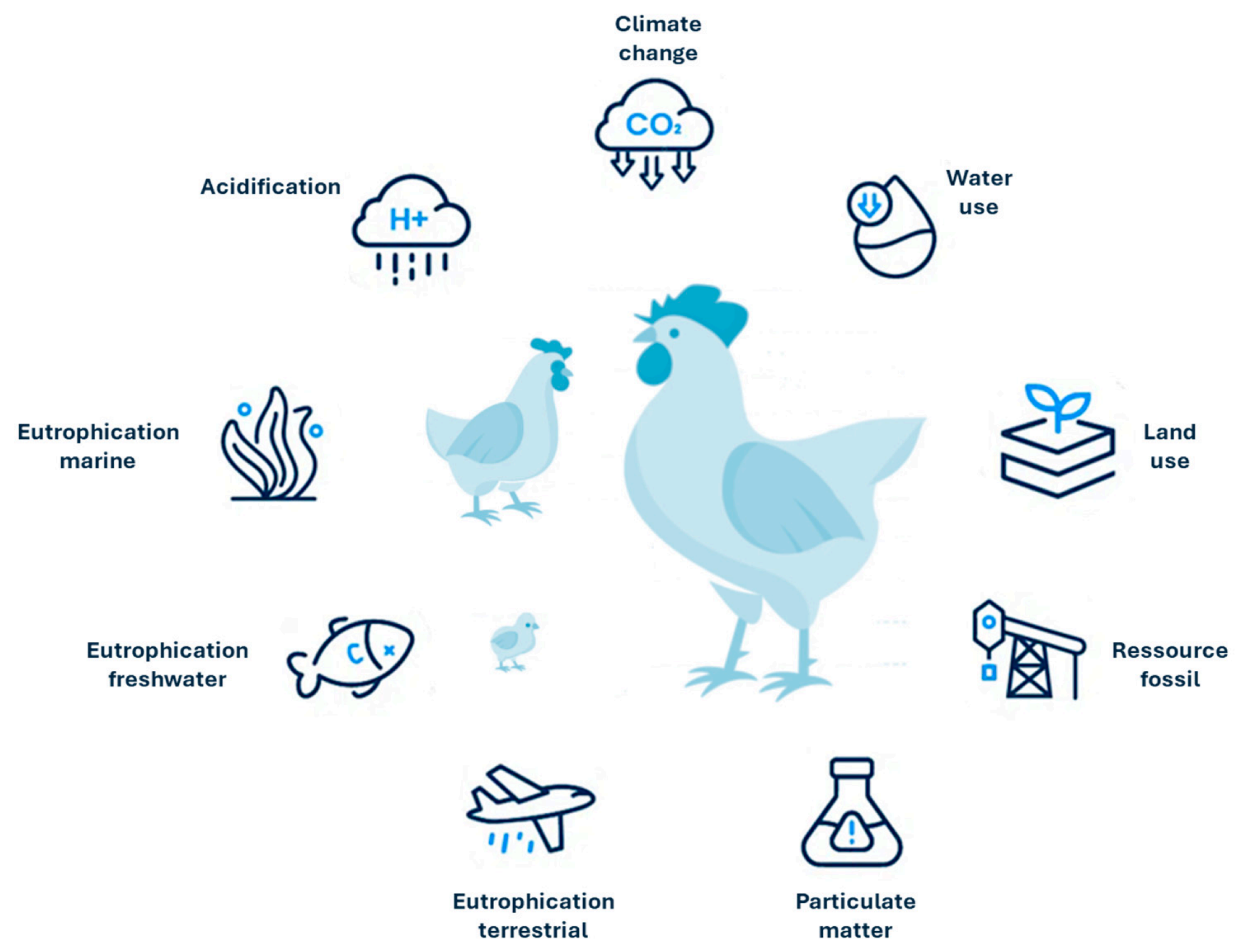
Environmental impact categories in broiler production evaluated in this LCA based on ISO 14040 and 14044 guidelines.

The life cycle analysis considered a selection of environmental impact categories, including total and disaggregated climate change (fossil, biogenic, and land use), land use, water use, acidification, eutrophication (at marine, freshwater and terrestrial levels), particulate matter formation, and fossil fuel abiotic depletion (Figure 2). These categories are illustrated in Figure 3.

The LCIA method followed the recommendations of the European Commission’s Product Environmental Footprint (PEF) framework, specifically using the Product Environmental Footprint Category Rules (PEFCRs) [33] for poultry production and animal feed [41], as well as the Food and Agriculture Organization (FAO) of the United Nations Livestock Environmental Assessment and Performance Partnership (LEAP) guidelines for feed additives in the livestock supply chain [42].

### 2.7. Broiler Chicken Performance Data Collection

Diets were formulated according to standard nutritional recommendations for the respective broiler genotypes and countries in which the trials were conducted, with the aim of meeting or slightly exceeding the animals’ requirements for crude protein and metabolizable energy [43]. The feed formulation was adapted to local practices while ensuring nutritional adequacy and consistency across treatments, in line with established broiler nutrition guidelines (Appendix A.1, Table A1).

Body weights were recorded upon arrival and subsequently at weekly intervals until 42 days of age (1386 birds per group). Weekly BWG and FI were calculated as the differences between initial and final body weights, and between feed offered and feed residuals, respectively. Weekly FI was then divided by the number of birds per replicate, after adjustment for mortality, to obtain the average FI per bird. The FCR was determined as the ratio of weekly FI to weekly BWG, accounting for mortality. The European Production Efficiency Factor (EPEF) was calculated [19] according to the following formula:

EPEF = (livability × live body weight (kg)/(age in days × FCR) × 100

Mortality was recorded daily for each replicate, and cumulative mortality rates were calculated. Zootechnical performance metrics such as FI, FCR, and BWG were derived from separate trials conducted in multiple countries. These trials compared broilers challenged with *Cl. perfringens* or exposed to heat stress, with or without *S. cerevisiae* supplementation. Trial-level data, including feed intake, weight gain, and mortality, are presented in Appendix A.1, Table A2. All environmental and zootechnical results are normalized per kg of LWG to eliminate bias from trial size differences.

Temperature management differed according to the experimental challenge. For trials conducted under *Cl. perfringens* challenge, birds were maintained under thermoneutral conditions (22–24 °C constant) throughout the 42-day cycle. For trials assessing heat stress, birds were exposed to cyclic heat stress protocols representative of commercial production in tropical and subtropical regions: ambient temperature was elevated to 28–36 °C during the warmest hours of the day (approximately 10:00–18:00), while nighttime temperatures were allowed to decrease to 24–28 °C. Relative humidity ranged from 55% to 80% depending on location. Ventilation systems (tunnel or cross-ventilation) were supplemented with evaporative cooling pads or foggers to mitigate extreme heat while maintaining the stress challenge. These conditions are consistent with published protocols for inducing heat stress in broiler research [44,45].

### 2.8. Sensitivity Analysis

**Figure A1:**
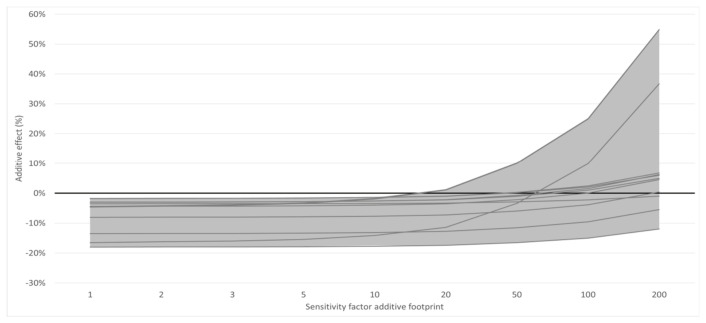
Sensitivity analysis to evaluate the relative impact of using proxy values for the production emissions of the additives on this study’s findings.

Since the objective of this study was to compare broiler production systems with and without feed additive supplementation, changes in the values of the assumed parameters were considered to have no significant influence on the conclusions, as such changes would apply equally in both systems. A sensitivity analysis was carried out to explicitly assess the contribution of additive production within the overall life cycle of the system under study (Figure A1). The analysis assessed the point at which the environmental impact of additive production could offset the environmental benefits associated with its use. A sensitivity analysis carried out by Blonk Consultants showed that the overall environmental impact of the additives remains stable as long as their emission factor does not exceed a critical threshold (“tipping point”) [40]. This finding confirms that using a proxy factor to estimate the carbon footprint of the additives is appropriate and does not significantly affect the results of the life cycle assessment. Variations in the proxy emission factor for additive production (±50%) altered the overall carbon footprint by <0.2%, confirming the robustness of our conclusions and supporting the use of the proxy factor.

## 3. Results

The results of the LCA were based on six studies conducted in different countries (USA, Brazil, Pakistan, and Mexico) under different management and challenging conditions (heat stress or *Cl. Perfringens* challenge). Each study compared the supplementation of *S. cerevisiae* (at 250–500 g per ton of feed) for 42 days with a positive control (PC).

### 3.1. Global Warming Potential and Environmental Impact

**Figure 4:**
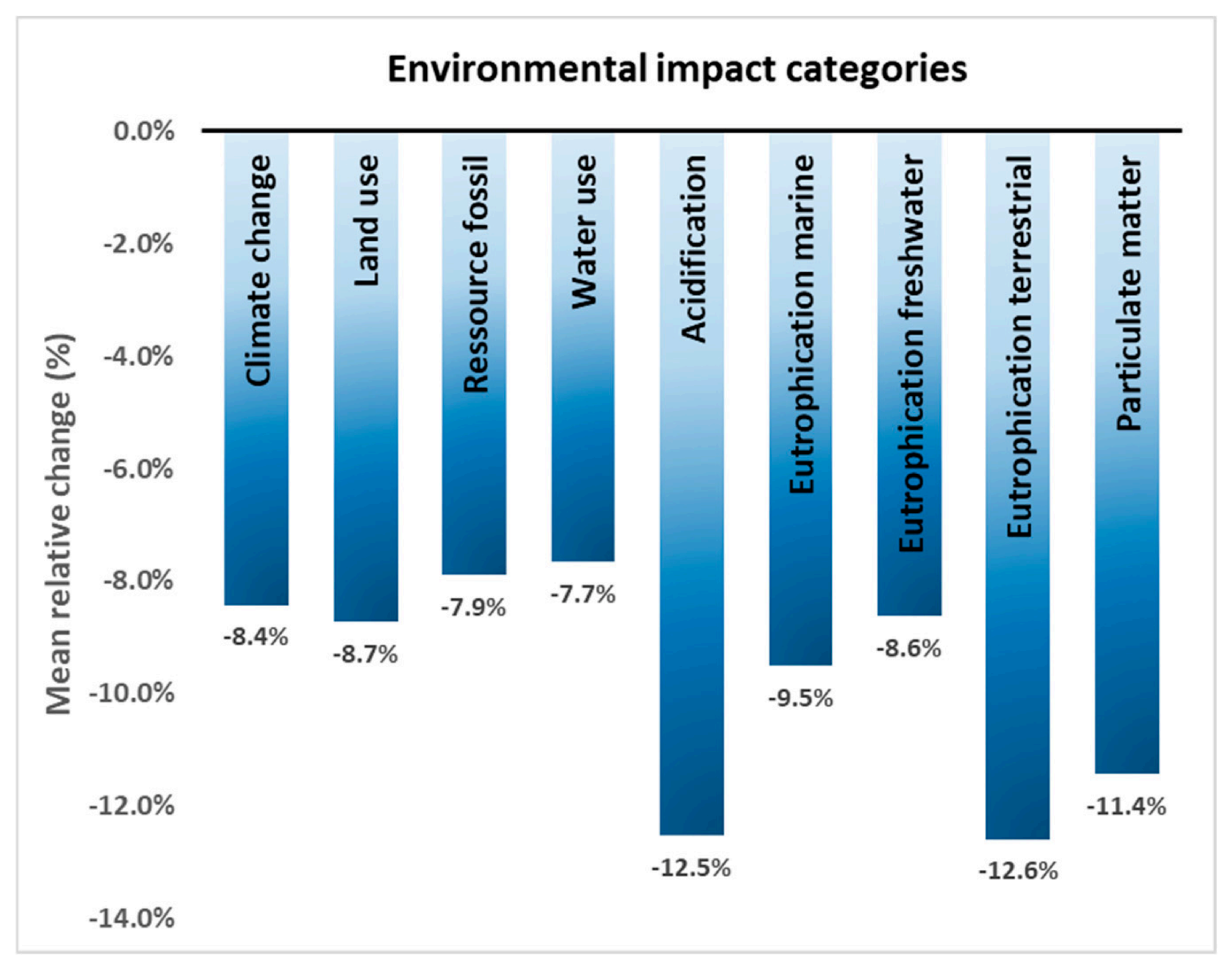
Mean relative change (%) in selected environmental categories associated with *S. cerevisiae* use for broiler feeding.

The mean environmental impact and resource use of the production systems are detailed in Table 1. Overall, the supplementation of *S. cerevisiae* (additive: AD) reduced climate change compared with PC by 0.19 kg CO_2_/kg of live weight, corresponding to an 8.4% reduction. The environmental impact of *S. cerevisiae* was lower than that of the PC in all the assessed categories. During the supplementation period, a beneficial effect was observed for land use (−8.7%), water use (−7.7%), resource use (−7.9%), acidification (−12.5%), marine eutrophication (−9.5%), freshwater eutrophication (−8.6%), terrestrial eutrophication (−12.6%), and particulate matter (−11.4%; Figure 4).

### 3.2. Impact of S. cerevisiae on Carbon Footprint Domains

**Figure 5:**
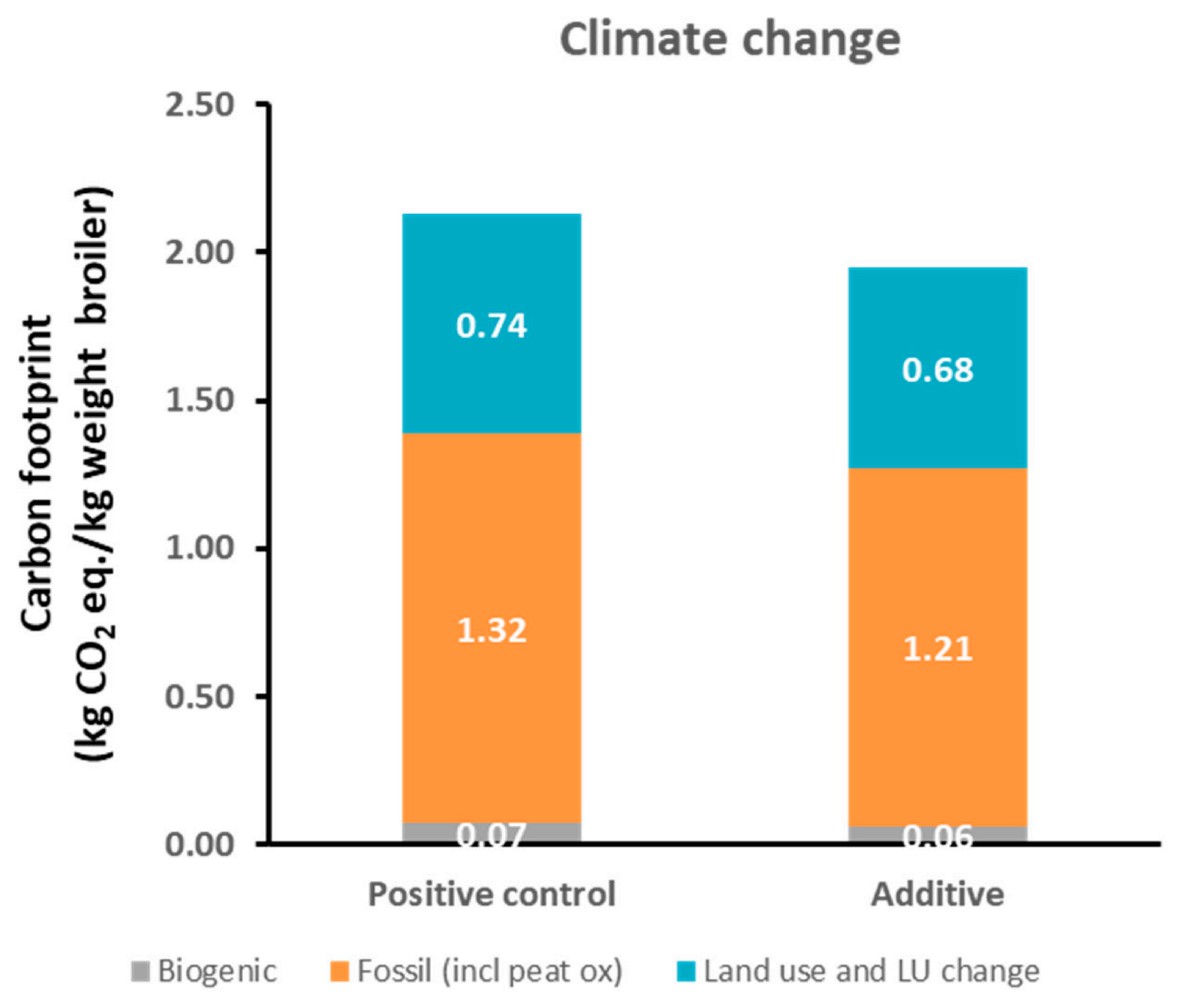
Climate change in selected environmental categories associated with *S. cerevisiae* use for broiler feeding. Abbreviations: CO_2_: carbon dioxide, eq: equivalent, kg: kilogram, LU: land use, and Ox: oxidation.

Climate change impacts can be divided into three domains: land use (LU) and land-use change (LUC), fossil, and biogenic carbon footprint. The average footprint of broilers supplemented with *S. cerevisiae* was lower than PC, with a reduction in biogenic carbon use by 14.3%, fossil fuel use by 8.3%, and LUC by 8.1% (Table 2 and Figure 5).

### 3.3. Contribution Analysis by Geographical Region

**Figure 6:**
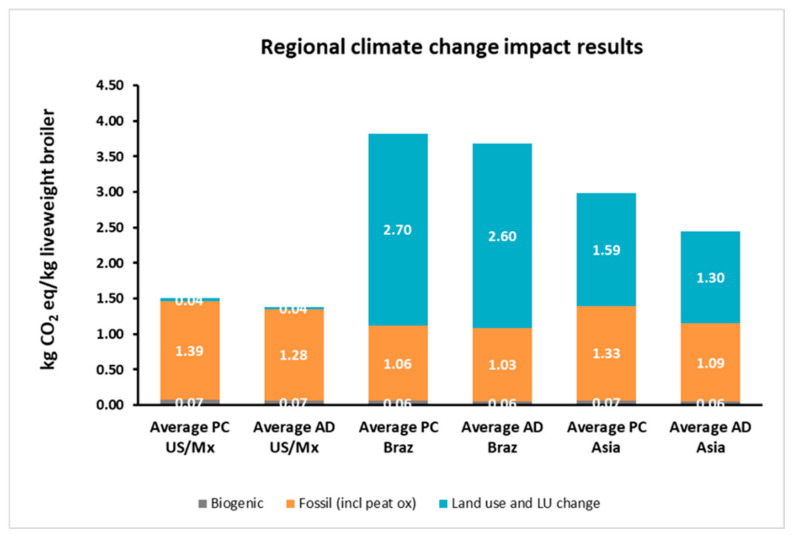
Climate change in selected environmental categories for broiler chicken feed with basic diet (PC) or supplemented with *S. cerevisiae* (AD). Abbreviations: AD: additive, Braz: Brazil, CO_2_: carbon dioxide, kg: kilogram, LU: land use, Ox: oxidation, and PC: positive control.

The overall climate change impact varied according to geographical location and management systems (Figure 6). The environmental impacts of the six trials were analyzed according to their region: US/Mexico (*n* = 4 studies), Brazil (*n* = 1 study), and Asia (*n* = 1 study).

For the US/Mexico trials, overall climate change was mainly driven by feed production (fossil use), with a contribution percentage of 68%. Supplementation with *S. cerevisiae* reduced the carbon footprint by 0.11 kg CO_2_ eq/kg of live weight (7.7%). For the trial conducted in Brazil, LUC was the main category contributing to the potential impacts (71%), followed by fossil fuel use (28%). Supplementation reduced the carbon footprint by 0.10 kg CO_2_ eq/kg (3.7%) due to LUC and 0.03 kg CO_2_ eq/kg (3.2%) due to fossil fuel use. For the trial conducted in Asia, LUC and fossil fuel use contributed almost equally (53% and 44%, respectively), showing a reduction in the carbon footprint of 0.19 kg CO_2_ eq/kg (18.3%) and 0.24 kg CO_2_ eq/kg (17.6%) due to LUC and fossil fuel use, respectively.

**Figure 7:**
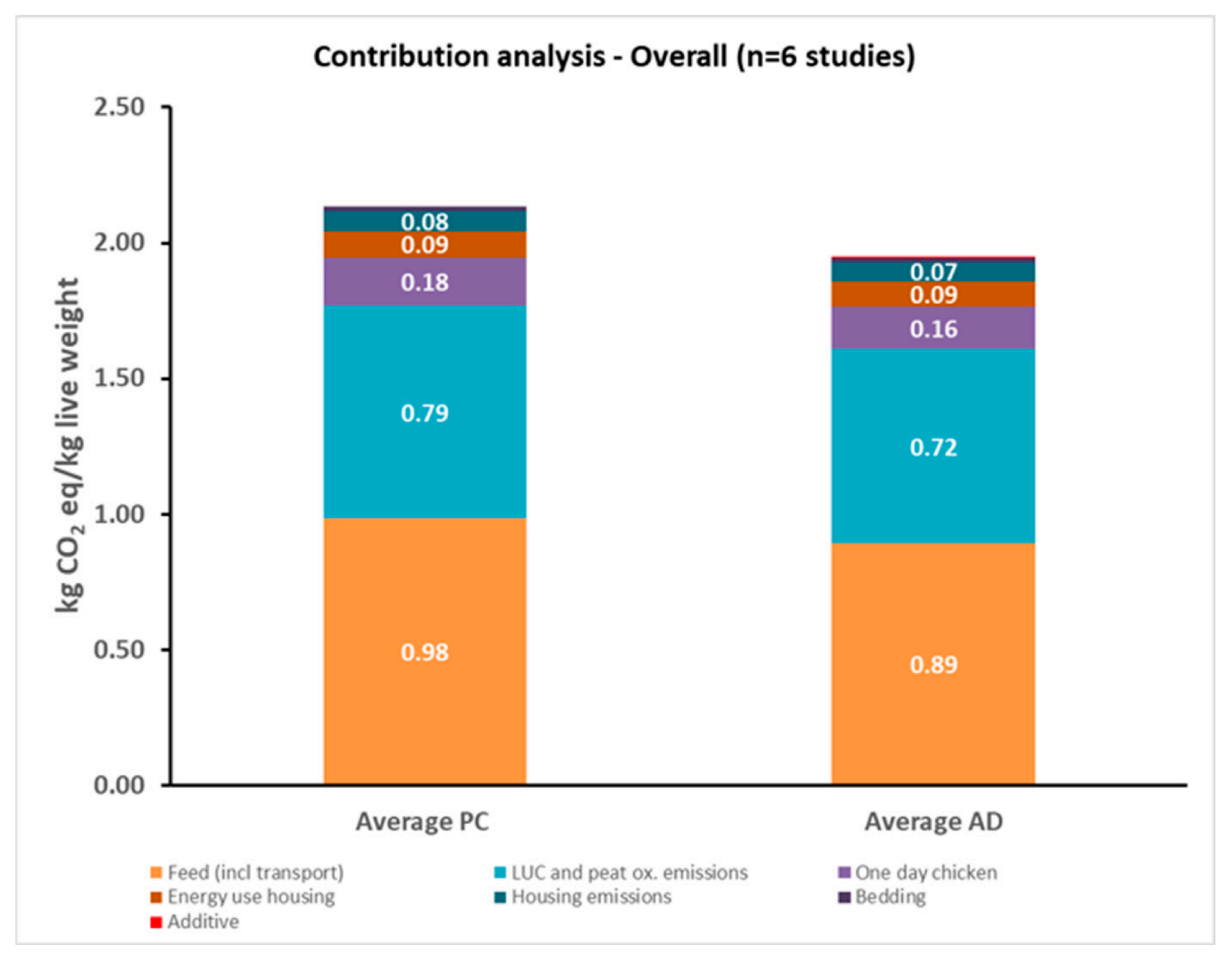
Contribution analysis to the carbon footprint of 1 kg of live weight broiler chicken supplemented or not with *S. cerevisiae*. Abbreviations: AD: additive, CO_2_: carbon dioxide, kg: kilogram, LU: land use, Ox: oxidation, and PC: positive control.

**Figure 8:**
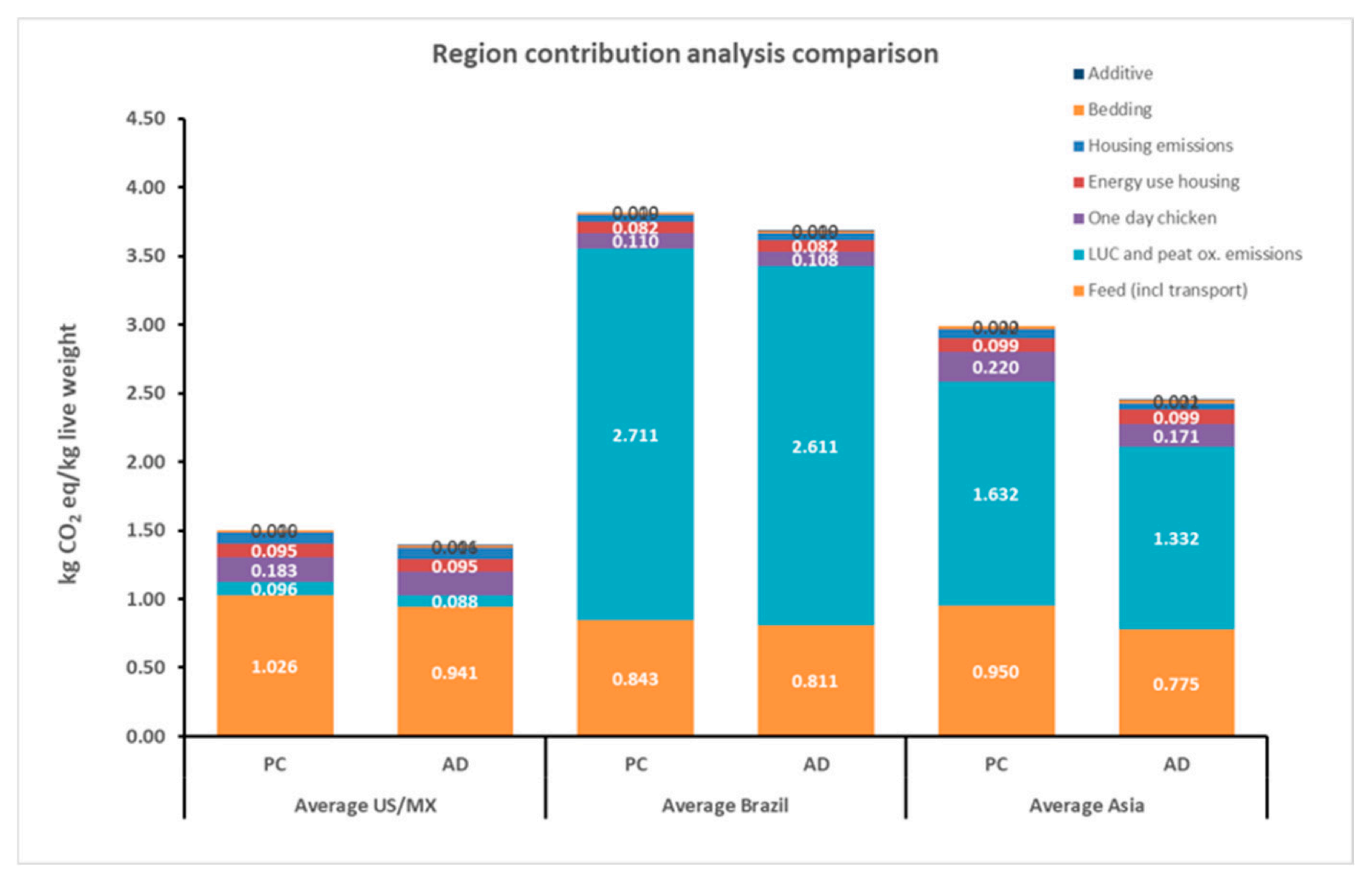
Contribution analysis to the carbon footprint according to geographical location. Abbreviations: AD: additive; PC: positive control.

The main contributors to the overall environmental benefits resulting from *S. cerevisiae* supplementation were feed, including transport (45.8%) and LUC (36.8%) (Figure 7). These proportions varied as a function of the production system’s location (Figure 8).

### 3.4. Broiler Chicken Performance

**Figure 9:**
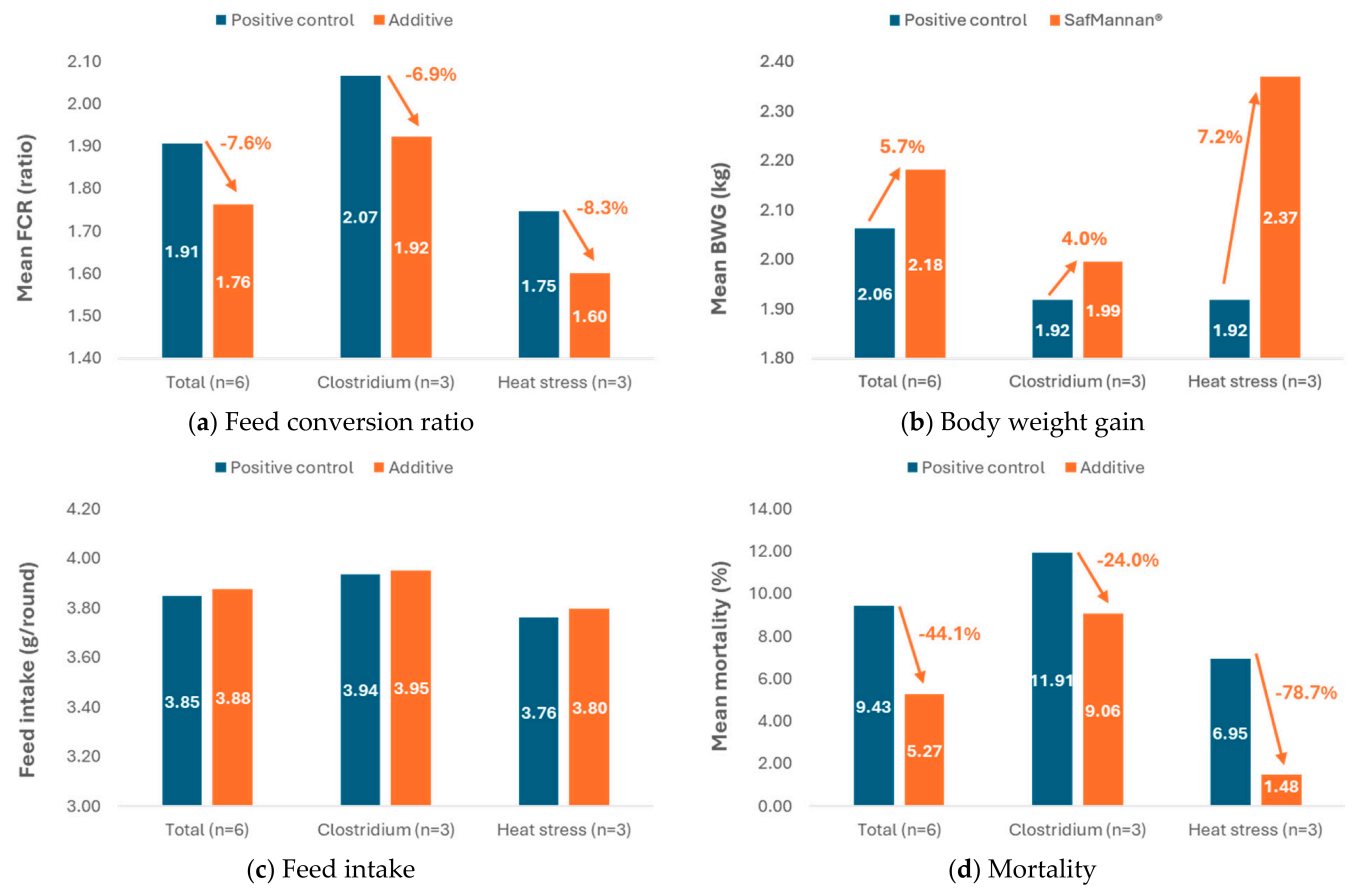
Effect of dietary *S. cerevisiae* (orange bar) supplementation on broiler chickens challenged with *Clostridium* or exposed to heat stress: (**a**) Feed conversion ratio (FCR); (**b**) body weight gain (BWG); (**c**) feed intake; and (**d**) mortality.

Overall, chickens supplemented with *S. cerevisiae* exhibited a lower FCR of 7.6% (Figure 9a), an increase in BWG of 5.7% (Figure 9b), and a decrease in mortality of 44.1% (Figure 9d) compared with the positive control. Feed intake was similar in both groups (Figure 9c).

Challenged chickens were analyzed separately, showing similar trends. The supplementation of *S. cerevisiae* decreased the FCR by 6.9% and 8.3% and increased BWG by 4.0% and 7.2% in chickens challenged with *Clostridium* or exposed to heat stress, respectively. A greater reduction in mortality was observed in chickens exposed to heat stress (−78.7%) than in those challenged with *Clostridium* (−24.0%).

## 4. Discussion

This study aimed to determine whether supplementing broiler diets with a yeast-derived postbiotic (*Saccharomyces cerevisiae*) could mitigate the environmental impact of production systems under stress conditions, specifically heat stress and *Clostridium perfringens* challenge. Our findings demonstrate that postbiotic supplementation not only enhances zootechnical performance but also significantly reduces the carbon footprint and other environmental indicators across diverse geographical regions. Overall, the trials achieved an 8.4% reduction in carbon footprint, equivalent to 0.19 kg CO_2_ eq per kg of live weight broiler output, with additional environmental impact categories showing improvements ranging from 7.7% to 12.6%. These results confirm that the use of yeast cell wall fractions in broiler production systems under challenging conditions offers a viable strategy to improve both productivity and environmental sustainability.

The contribution analysis showed different results depending on geographical location. Feed accounted for the majority of GHG emissions in North America (68%, corresponding to 1.39 kg CO_2_ eq per kg of live weight), while LUC was the main contributor in Brazil (71%, corresponding to 2.70 kg CO_2_ eq per kg of live weight). This is in line with other studies reporting that feed provision accounted for 82% of GHG emissions in US broiler production [46], and the spread of land used for soybean cultivation in Brazil associated with LUC emissions from deforestation [47]. We showed that *S. cerevisiae* supplementation reduced overall GHG emissions by 7.7%, 3.5% and 18% in North America, Brazil and Asia, respectively. The positive effect of *S. cerevisiae* supplementation on carbon footprint caused by feed and LUC is closely related to the improvement of FCR. These findings align with previous LCA studies on the same yeast probiotic in ruminants, which reported a 2.9–5.0% reduction in the carbon footprint for dairy cows [30] and a 3.8–6.6% reduction for beef cattle [31]. This confirms that the improvement in feed efficiency is a consistent and reliable driver of environmental benefits across different livestock species. A lower FCR reduces GHG emissions since the requirement for feed, particularly imported crops (soybeans), is downsized. In a study in which antibiotics were replaced by *S. cerevisiae* mixed in feed for broiler chicken, the results showed improved digestion of crude proteins, fat, and fiber, and better absorption of nitrogen, calcium and phosphorus [22]. This better nutrient assimilation is due to the MOS- and beta-glucans-rich composition in the YCW extract, which have already been demonstrated to optimize nutrient assimilation. In a recent meta-analysis, they reported that the use of MOSs as an alternative to antibiotic growth promoters led to a 3.7% increase in BWG and 3.0% decrease in the FCR [48]. In this LCA study, supplementation of *S. cerevisiae* to the basic diet showed a 5.7% increase in BWG and a 7.6% decrease in FCR. Moreover, studies showed that supplementation of yeast hydrolysate linearly reduced ammonia emissions by up to 10% in broiler houses [49,50]. Ammonia itself is not a GHG but is considered a major precursor through indirect N_2_O emissions, with a global warming potential of nearly 300 times that of CO_2_ over a 100-year timescale [39].

In addition to the nutritional benefits of MOSs, several studies have reported antioxidant properties, demonstrating their role as free radical scavengers and reducing oxidative damage induced by heat stress [51,52]. Heat stress is particularly serious in poultry production systems located in tropical or subtropical regions [44], where the impact of climate change could lead to an increase in outdoor land surface temperature of 2–6 °C by the end of the 21st century, as projected for West Africa, for instance [53]. One study showed that broilers exposed to chronic heat stress experienced negative impacts on the FCR (25.6%), BW (−32.6%), and mortality (79.9%), and that dietary supplementation with MOSs can partially reduce these detrimental effects [45]. In the present study, supplementation of *S. cerevisiae* in the feed of chickens exposed to heat stress resulted in improvements in the FCR (−8.3%), BW (7.2%), and mortality (−78.7%). In heat stress, the intestinal barrier is compromised by affecting gut structure [54,55] and enhancing facultative anaerobiotic bacteria [56] and pathogen proliferation [54], such as *E. coli* [57] or *Cl. Perfringens* [12]. However, the growth of *E. coli* causing colibacillosis can be inhibited by the use of MOSs due to their ability to prevent bacterial adhesion to epithelial cells [17,58]. Specifically, direct binding capacity to *E. coli* was demonstrated by scanning electron microscopy [59], while studies have validated its efficacy against poultry-derived Enterotoxigenic *E.coli* (ETEC) isolates [58,60]. Beyond direct binding, supplementation of *S. cerevisiae* modulates intestinal inflammation and gut barrier integrity by downregulating the expression of TLR4 and NF-κB pathways, as well as reducing serum levels of endotoxin and diamine oxidase in challenged birds with *E. coli* [61]. Consequently, field data indicate a rapid reduction in mortality within 48 h following supplementation during *E. coli* outbreaks, even without antibiotic use [58].

*C. perfringens* is the main causative pathogen of NE in poultry. NE is recognized as a major intestinal diseases in poultry production, affecting an estimated 40% of commercial poultry farms, with daily mortality rates reaching up to 1% and resulting in considerable economic losses [62]. Approximately 75 to 95% of broiler chickens have *C. perfringens* as part of their normal intestinal microflora [63]. Factors contributing to its development are multiple, such as environmental contamination, concurrent infections or gut microbiota dysbiosis [62,64]. Alternative additives, including probiotics and prebiotics, to replace antibiotics have been studied [65]. MOSs have been shown to reduce bacterial invasion and decrease mortality induced by NE [66]. When challenged with *C. perfringens*, chickens had a higher FCR and a lower BW than unchallenged birds. In this study, FCR and BWG showed improvements of 6.9% and 4%, respectively, with a reduction in mortality of 24.0% when *S. cerevisiae* was mixed into feed. This is in accordance with previous studies showing that YCW improved performance [18,48]. However, other studies reported little effect of YCW on growth performance, probably due to the intensity of NE challenge used [66,67].

The robustness of this study’s results was supported by the consistent use of primary data, sensitivity analysis, and the negligible contribution of additive production to the overall environmental footprint of broilers. These findings position *S. cerevisiae* as an effective tool for improving environmental sustainability in broiler production systems.

## 5. Conclusions

This LCA demonstrates that supplementing broiler diets with a yeast-derived postbiotic from *S. cerevisiae* can substantially reduce the environmental footprint of broiler production from cradle to farm gate. Across six trials conducted on three continents and under two challenging conditions (heat stress and *Clostridium perfringens* challenge), *S. cerevisiae* supplementation reduced the climate change impact by 8.4%, corresponding to −0.19 kg CO_2_ eq per kg of live weight, and consistently decreased additional environmental indicators, including land use (−8.7%), water scarcity (−7.7%), resource use (−7.9%), acidification (−12.5%), marine eutrophication (−9.5%), freshwater eutrophication (−8.6%), terrestrial eutrophication (−12.6%), and particulate matter formation (−11.4%).

These environmental improvements were associated with enhanced zootechnical performance, characterized by a 7.6% reduction in the FCR, a 5.7% increase in BW, and a 44.1% reduction in mortality, even under stress conditions. Taken together, these results indicate that the observed sustainability gains are mainly driven by improved biological efficiency and animal resilience. Overall, this study supports that the yeast cell wall fraction is a practical and scalable nutritional strategy that can simultaneously improve productivity and reduce environmental burdens in conventional broiler production systems across various geographical and management contexts. Future research is required to focus on validating these results across a broader range of production systems and geographies, as well as assessing the potential long-term benefits of *S. cerevisiae* supplementation, including its impact on bird health and litter quality. The limitations of this study include the use of proxy data for additive production and the reliance on specific regional databases, which may not capture all local variability in commercial farms. Future LCA studies should aim to incorporate primary data for additive manufacturing and expand the geographical scope to include diverse commercial farming practices.

## Figures and Tables

**Table 1:** Environmental impact of *S. cerevisiae* supplementation.

Impact Category	Unit	Positive Control	Additive
Climate change	kg CO_2_ eq	2.14	1.95
Acidification	mol H^+^ eq	0.06	0.05
Land use	Pt	256.37	234.78
Resource use, fossils	MJ	13.38	12.25
Water scarcity	m^3^ depriv.	2.93	2.68
Eutrophication, marine	kg N eq	1.11 × 10^−2^	1.00 × 10^−2^
Eutrophication, freshwater	kg P eq	8.87 × 10^−4^	8.11 × 10^−4^
Eutrophication, terrestrial	mol N eq	0.27	0.24
Particulate matter	disease inc.	5.30 × 10^−7^	4.65 × 10^−7^

Abbreviations: CO_2_: carbon dioxide; eq: equivalent; mol: mole; H^+^: hydrogen proton; m^3^: cubic meter; MJ: mega joules; N: nitrogen; P: phosphorus; Pt: point.

**Table 2:** Comparison of carbon emissions in selected environmental categories from broiler production with or without *S. cerevisiae* supplementation.

Carbon Footprint (kg CO_2_ eq)	Positive Control	Additive	Difference (%)
Climate change–biogenic	0.07	0.06	−14.3%
Climate change–fossil (incl. peat ox)	1.32	1.21	−8.3%
Climate change–LU and LUC	0.74	0.68	−8.1%

Values are presented as kg CO_2_ eq/kg weight of broiler. *S. cerevisiae* CO_2_: carbon dioxide, LU: land use, LUC: land-use change, and Ox: oxidation.

**Figure A2:**
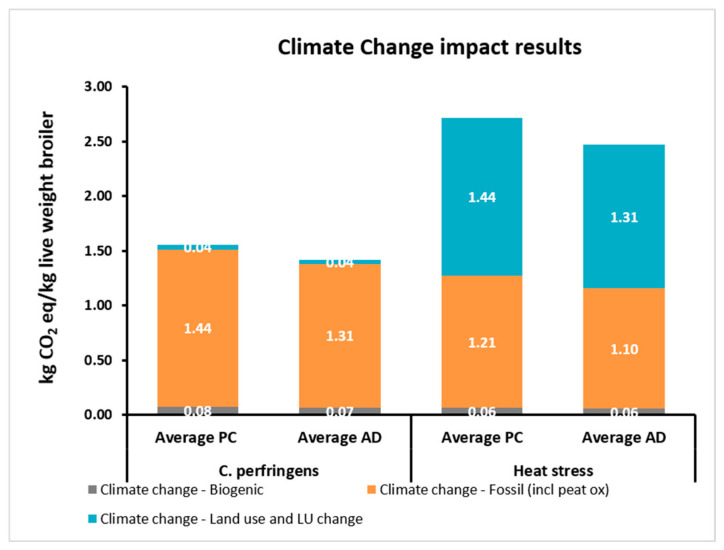
Climate change in selected environmental categories associated with *S. cerevisiae* use for broiler feeding according to conditions: challenged with *Cl. Perfringens* or exposed to heat stress. Abbreviations: AD: additive; PC: positive control.

## Data Availability

The original contributions presented in this study are included in this article. Further inquiries can be directed to the corresponding author.

## Abbreviations

The following abbreviations are used in this manuscript:

AD	Additive
BW	Body Weight
BWG	Body Weight Gain
CFU	Colony-Forming Unit
CH_4_	Methane
CO_2_	Carbon Dioxide
EEA	European Environment Agency
EMEP	European Monitoring and Evaluation Program
EPEF	European Production Efficiency Factor
eq	Equivalent
FAO	Food and Agriculture Organization
FCR	Feed Conversion Ratio
FI	Feed Intake
H+	Cation of Hydrogen or Proton
IPCC	Intergovernmental Panel on Climate Change
kg	Kilogram
LCA	Life Cycle Assessment
LCI	Life Cycle Inventory
LCIA	Life Cycle Impact Assessment
LEAP	Livestock Environmental Assessment and Performance Partnership
LU	Land Use
LUC	Land-Use Change
LWG	Live Weight Gain
MJ	Megajoules
MOSs	Mannan-Oligosaccharides
N	Azote
NH_3_	Ammonia
NMVOC	Non-Methane Volatile Organic Compound
NO_x_	Nitric Oxide
NO_2_	Nitrogen Dioxide
NO_x_	Nitrous Oxides
OECD	Organization for Economic Co-operation and Development
P	Phosphorus
PC	Positive Control
PEF	Product Environmental Footprint
PEFCRs	Product Environmental Footprint Category Rules
YCW	Yeast Cell Wall

## Appendix A

### Appendix A.1. Tables

**Table A1:** Broiler chicken diet for each trial.

Ingredients	TRIAL 1			Ingredients	TRIAL 2			Ingredients	TRIAL 3		
(Amount kg/ton)	Starter	Grower	Finisher	(Amount kg/ton)	Starter	Grower	Finisher	(Amount kg/ton)	Starter	Grower	Finisher
Corn	668.4	673.2	659.8	Corn	528.6	585.6	636.7	Corn	668.4	673.2	659.8
Soybean meal	228.9	182.7	151.1	Soybean meal	398.4	333.7	287.0	Soybean meal	228.9	182.7	151.1
Meat & Bone meal	40.0	40.0	40.0	Vegetable fat	31.4	41.5	42.6	Meat & Bone meal	40.0	40.0	40.0
Soybean oil	0.0	4.2	13.0	Limestone	11.2	10.0	8.0	Limestone	4.7	4.5	4.2
Limestone	4.7	4.5	4.2	Dicalcium phosphate	20.3	18.3	15.4	Dicalcium phosphate	4.4	2.7	0.9
Dicalcium phosphate	4.4	2.7	0.9	Salt	3.8	3.9	3.9	Salt	4.5	4.3	4.2
Salt	4.5	4.3	4.2	L-lysine	0.2	1.5	1.2	Choline	0.8	0.8	0.8
Choline	0.8	0.8	0.8	DL-methionine	2.8	2.3	2.0	Vitamin premix	0.4	0.4	0.4
L-lysine	3.3	3.4	3.0	Vitamin premix	2.5	2.5	2.5	Trace mineral	0.9	0.9	0.7
DL methionine	2.8	2.2	1.6	Trace mineral	0.8	0.7	0.7	L-lysine	3.3	3.4	3.0
L-threonine	0.8	0.7	0.3					DL methionine	2.8	2.2	1.6
Vitamin premix	1.4	1.3	1.1					L-threonine	0.8	0.7	0.3
Phytase (DDGS)	40.0	80.0	120.0					Phytase (DDGS)	40.0	80.0	120.0
								Phytase (quantum blue)	0.2	0.2	0.2
								Soybean oil		4.2	13.0
Ingredients	TRIAL 4			Ingredients	TRIAL 5			Ingredients	TRIAL 6		
(Amount kg/ton)	Starter	Grower	Finisher	(Amount kg/ton)	Starter	Grower	Finisher	(Amount kg/ton)	Starter	Grower	Finisher
Corn	570.0	632.7		Corn	584.1	612.8		White corn 8.5%	551.1		
Soybean meal	323.0	270.0		Soybean meal	344.4	299.4		Soybean meal 46%	365.0		
Soybean oil	32.7	36.0		Blended fat	28	47.99		Soybean oil	31.0		
Meat meal	59.5	48.0		Limestone	15.7	15.8		Dicalcium phosphate 21/18	19.4		
Limestone	1.3	0.7		Monocalcium phosphate 16/21P	15.6	14.1		Limestone	16.2		
Salt	3.5	3.7		Sodium chloride	4.1	3		Salt	3.1		
Choline	0.3	0.5		L-lysine	1.6	1.3		Methionine hydroxy analog	4.0		
L-lysine	1.7	1.9		DL methionine	1.5	1.5		L-lysine	2.7		
DL methionine	3.3	2.3		L-threonine	0.3	0.3		Sodium bicarbonate	2.2		
L-threonine	0.7	0.2		Salinomicyn	0.0	0.5		Vitamins + minerals + choline premix	4.0		
Vitamin premix	4.0	4.0		Vitamin premix	2.5	2.5		Nicarbazin	0.5		
				Trace mineral	0.5	0.5		Salinomicyn	0.0		
								L-threonine	0.7		
								Antioxidant	0.2		
								Pigment 20 g/k	0.0		
								Canthaxanthin-based pigment 1%	0.0		

Trials 4 and 5: Starter and grower diets were fed from 0 to 21 and 22 to 42 days of age, respectively. In Trial 6, the starter diet was provided throughout the entire experimental period (0–42 days). All diets were formulated to meet or slightly exceed the birds’ requirements for crude protein and metabolizable energy [32].

**Table A2:** Zootechnical description.

Breeds	ROSS 308											
Challenge	*Clostridium*						Heat stress					
Trial Name	Trial 1		Trial 2		Trial 3		Trial 4		Trial 5		Trial 6	
Geography	Virginia, USA		Georgia, USA		Virginia, USA		Brazil		Pakistan		Mexico	
Year	2017		2018		2018		2016		2012		2015	
Length of a round (days)	42		42		42		42		42		42	
Yeast cell wall fraction (g/t of feed)	250		250		250		250		500		250	
Group	PC	AD	PC	AD	PC	AD	PC	AD	PC	AD	PC	AD
Output data												
Total broilers to slaughter (liveweight kg/round)	637.2	683.64	1115	1200	397.52	454.98	347.1	354.24	131.73	169.7	33.76	35.97
Housing systems												
Purchased one day chicken (n/round)	360	360	500	500	300	300	120	120	90	90	16	16
BW (kg)	1.77	1.899	2.23	2.4	1.7513	1.685	2.892	2.952	1.626	1.907	2.11	2.248
MR (% on a round)	11.38	4.17	8.8	13	15.56	10	10.84	3.34	10	1.11	0	0
FCR	2.103	1.913	1.86	1.82	2.234	2.034	1.57	1.51	1.67	1.39	1.996	1.902
MAFCR	2.016	1.899	1.81	1.73	2.059	1.932	*	*	*	*	1.996	1.902
Starter (kg/round)	189.51	187.96	229.4	229.5	152.1	131.27	148.05	147.15	80.87	88.16	65.94	67.04
Grower (kg/round)	462.88	459.77	1263.9	1325.3	351	326.24	396.8	387.8	144.2	148.43		
Finisher (kg/round)	677.30	649.54	810	858.7		570.74						

Abbreviations: AD: additive; BW: body weight; FCR: feed conversion ratio; MR: mortality rate; MAFCR: mortality-adjusted feed conversion ratio; PC: positive control. * In trials 4 and 5, MAFCR was not reported since the standard FRC was already dynamically adjusted for daily mortality.

### Appendix A.2. Figures

For this sensitivity analysis, an incremental increase in the assumed carbon footprint of the feed additives was evaluated in relation to the carbon footprint reduction achieved by their use. For each intervention scenario, the contribution of the additive was multiplied by progressively larger sensitivity factors. Each line corresponds to a distinct intervention or condition affecting the environmental performance of the additives. The shaded grey area indicates the range of intervention effects. A steeper rising line represents a scenario in which the additive contributes more substantially to the baseline carbon footprint.

Only when the emission factor surpasses a certain threshold—approximately fifteen times the proxy carbon footprint of the additives—do some scenarios demonstrate a net increase in carbon footprint following intervention. When the emission factor is altered by less than a factor of ten, the intervention effect of the additives remains essentially unchanged. Therefore, it can be concluded that even if the proxy carbon footprint underestimated the actual production emissions of the feed additives, it would need to be underestimated by at least a factor of ten to result in a net increase in the carbon footprint of the intervention scenarios.

## References

[1] Dittoe D., Ricke S.C., Kiess A., Ricke S.C. (2020). Commercial poultry production and gut function—Historical perspective. Improving Gut Health in Poultry.

[2] OECD, FAO (2025). OECD-FAO Agricultural Outlook 2025–2034.

[3] Ben Sassi N., Averos X., Estevez I. (2016). Technology and Poultry Welfare. Animals.

[4] Oke O.E., Akosile O.A., Uyanga V.A., Oke F.O., Oni A.I., Tona K., Onagbesan O.M. (2024). Climate change and broiler production. Vet. Med. Sci..

[5] Costantini M., Ferrante V., Guarino M., Bacenetti J. (2021). Environmental sustainability assessment of poultry productions through life cycle approaches: A critical review. Trends Food Sci. Technol..

[6] Kpomasse C.C., Oke O.E., Houndonougbo F.M., Tona K. (2021). Broiler production challenges in the tropics: A review. Vet. Med. Sci..

[7] Lara L.J., Rostagno M.H. (2013). Impact of Heat Stress on Poultry Production. Animals.

[8] Chauhan S.S., Rashamol V.P., Bagath M., Sejian V., Dunshea F.R. (2021). Impacts of heat stress on immune responses and oxidative stress in farm animals and nutritional strategies for amelioration. Int. J. Biometeorol..

[9] Apalowo O.O., Ekunseitan D.A., Fasina Y.O. (2024). Impact of Heat Stress on Broiler Chicken Production. Poultry.

[10] Liu L., Ren M., Ren K., Jin Y., Yan M. (2020). Heat stress impacts on broiler performance: A systematic review and meta-analysis. Poult. Sci..

[11] Lauridsen C. (2019). From oxidative stress to inflammation: Redox balance and immune system. Poult. Sci..

[12] Tsiouris V., Georgopoulou I., Batzios C., Pappaioannou N., Ducatelle R., Fortomaris P. (2018). Heat stress as a predisposing factor for necrotic enteritis in broiler chicks. Avian Pathol..

[13] Van Boeckel T.P., Brower C., Gilbert M., Grenfell B.T., Levin S.A., Robinson T.P., Teillant A., Laxminarayan R. (2015). Global trends in antimicrobial use in food animals. Proc. Natl. Acad. Sci. USA.

[14] Urban J., Kareem K.Y., Atanasov A.G., Matuszewski A., Bień D., Ciborowska P., Rygało-Galewska A., Michalczuk M. (2024). Postbiotics, a natural feed additive for growth performance, gut microbiota and quality of poultry products. Curr. Res. Biotechnol..

[15] Jahedi S., Pashangeh S. (2025). Bioactivities of postbiotics in food applications: A review. Iran. J. Microbiol..

[16] Roque F., Lopes M.H.S., Raffi P., Oliveira R., Caparroz M., Longhini G., Granghelli C., Faria D., Araujo L., Araujo C. (2025). Postbiotics: An Alternative for Improving Health and Performance of Poultry Production. Microorganisms.

[17] Asbury R.E., Saville B.A. (2025). Manno-oligosaccharides as a promising antimicrobial strategy: Pathogen inhibition and synergistic effects with antibiotics. Front. Microbiol..

[18] M’Sadeq S.A., Wu S.B., Choct M., Forder R., Swick R.A. (2015). Use of yeast cell wall extract as a tool to reduce the impact of necrotic enteritis in broilers. Poult. Sci..

[19] Zhong X., Wang G., Li F., Fang S., Zhou S., Ishiwata A., Tonevitsky A.G., Shkurnikov M., Cai H., Ding F. (2023). Immunomodulatory Effect and Biological Significance of beta-Glucans. Pharmaceutics.

[20] Halas V., Nochta I. (2012). Mannan Oligosaccharides in Nursery Pig Nutrition and Their Potential Mode of Action. Animals.

[21] Molina D., Marinas I.C., Angamarca E., Hanganu A., Stan M., Chifiriuc M.C., Tenea G.N. (2025). Postbiotic-Based Extracts from Native Probiotic Strains: A Promising Strategy for Food Preservation and Antimicrobial Defense. Antibiotics.

[22] Shatskikh E., Nufer A., Neverova O., Galiev D. (2019). Digestibility and nutrient absorption in broiler chickens when replacing feed antibiotics in mixed feed with safe growth promoters. Adv. Intell. Syst. Res..

[23] Mohammed Ali F.Q., Al Shukri A.Y. (2020). Effect of the use of Safmannan as a prebiotic in production and performance of broiler. Biochem. Cell. Arch..

[24] Ali N.A.-L., Jameel Y., Abbas R.J. (2019). Effects of Dietary Prebiotics (SafMannan) and Local Iraqi Prebiotic on Some Blood Biochemical Parameters of Broiler Chickens. Pak. J. Nutr..

[25] Jameel Y.J., Alrekabi M.M., Ali N.A. (2020). Effect of SafMannan on Local Iraqi Laying Hens Performance. Indian J. Forensic Med. Toxicol..

[26] Nuningtyas Y.F., Natsir M.H., Widodo E., Rahman M.M., Atole A.F.F., Utami M.M.D., Sjofjan O. (2026). Postbiotics in broiler nutrition: A critical review of mechanisms, efficacy, and applications in antibiotic-free production systems. Poult. Sci..

[27] Ogbuewu I., Okoro V., Mbajiorgu C. (2020). Probiotic-yeast improves performance indicators in broiler chickens: Evidence from meta-analysis. Appl. Ecol. Environ. Res..

[28] Liu Q., Ni X., Chen C., Xu J., Pei E., Yang A., Xu M., Wang X., Fu S., Yu R. (2025). Exploring the Impact of Dietary EPA/DHA Supplementation on Lipid Metabolism of Tenebrio molitor Larvae. Insects.

[29] Liu S., Wang K., Lin S., Zhang Z., Cheng M., Hu S., Hu H., Xiang J., Chen F., Li G. (2023). Comparison of the Effects between Tannins Extracted from Different Natural Plants on Growth Performance, Antioxidant Capacity, Immunity, and Intestinal Flora of Broiler Chickens. Antioxidants.

[30] Salah N., Legendre H., Paiva E., Duclos J., Briche M., Maaoui M., Scholten J., Garat Boute C. (2024). Quantification of the Environmental Impact of Feeding Yeast Probiotic *Saccharomyces* cerevisiae Actisaf Sc 47 in Dairy Cow: A Life Cycle Assessment Approach. Animals.

[31] Salah N., Legendre H., Paiva E., Duclos J., Briche M., Colbalchini F., Gac A., Kerihuel T., Boute C.G. (2024). Does the Use of the Yeast Probiotic *Saccharomyces* cerevisiae Actisaf Sc 47 Reduce the Environmental Impacts of Beef Cattle? A Study Based on Life Cycle Assessment. Animals.

[32] Tyszler H.B.M., Braconi M.V.P.N., van Rijn N.D.J. (2022). Agri-Footprint 6—Methodology Report.

[33] European Commission (2018). Product Environmental Footprint Category Rules.

[34] (2006). Environmental Management—Life Cycle Assessment—Principles and Framework.

[35] (2020). Environmental Management—Life Cycle Assessment—Requirements and Guidelines—Amendment 2.

[36] Xiang J., Chen S., Tan S. (2026). A comprehensive review of the Maillard reaction in solid pharmaceutical dosage forms: A focus on lactose. Expert Opin. Drug Deliv..

[37] IPCC (2006). Guidelines for National Greenhouse Gas Inventories.

[38] EMEP/EEA (2023). Air Pollutant Emission Inventory Guidebook 2023.

[39] IPCC (2021). Climate Change 2021: The Physical Science Basis.

[40] Blonk H., Bosch H., Braconi N., Van Cauwenberghe S., Kok B. (2021). The Applicability of LCA Guidelines to Model the Effects of Feed Additives on the Environmental Footprint of Animal Production.

[41] European Commission (2018). PEFCR Feed for Food Producing Animals.

[42] FAO (2020). Environmental Performance of Feed Additives in Livestock Supply Chains—Guidelines for Assessment.

[43] National Research Council (U.S.) (1994). Nutrient Requirements of Poultry.

[44] Renaudeau D., Collin A., Yahav S., de Basilio V., Gourdine J.L., Collier R.J. (2012). Adaptation to hot climate and strategies to alleviate heat stress in livestock production. Animal.

[45] Sohail M.U., Hume M.E., Byrd J.A., Nisbet D.J., Ijaz A., Sohail A., ShabbiRRr M.Z., Rehman H. (2012). Effect of supplementation of prebiotic mannan-oligosaccharides and probiotic mixture on growth performance of broilers subjected to chronic heat stress. Poult. Sci..

[46] Pelletier N. (2008). Environmental performance in the US broiler poultry sector: Life cycle energy use and greenhouse gas, ozone depleting, acidifying and eutrophying emissions. Agric. Syst..

[47] Caro D., Davis S.J., Kebreab E., Mitloehner F. (2018). Land-use change emissions from soybean feed embodied in Brazilian pork and poultry meat. J. Clean. Prod..

[48] Polidoro B.R., de Oliveira M.J.K., Braga F., Polycarpo G.D.V. (2024). Mannan oligosaccharide as an alternative to infeed antibiotics to improve growth performance of broilers: A meta-analysis. Br. Poult. Sci..

[49] Riaz R., Ölmez M., Karadağoğlu Ö., Şahin T., Kukovics S., James Grichar W. (2024). Effects of Natural Feed Additives on Reducing the Carbon Footprint in Broiler Farms. Animal Husbandry—Beliefs, Facts and Reality.

[50] Sampath V., Han K., Kim I.H. (2021). Influence of yeast hydrolysate supplement on growth performance, nutrient digestibility, microflora, gas emission, blood profile, and meat quality in broilers. J. Anim. Sci. Technol..

[51] Cheng Y., Du M., Xu Q., Chen Y., Wen C., Zhou Y. (2018). Dietary mannan oligosaccharide improves growth performance, muscle oxidative status, and meat quality in broilers under cyclic heat stress. J. Therm. Biol..

[52] Zheng C., Li F., Hao Z., Liu T. (2018). Effects of adding mannan oligosaccharides on digestibility and metabolism of nutrients, ruminal fermentation parameters, immunity, and antioxidant capacity of sheep. J. Anim. Sci..

[53] Sylla M.B., Nikiema P.M., Gibba P., Kebe I., Klutse N.A.B. (2016). Climate Change over West Africa: Recent Trends and Future Projections. Adaptation to Climate Changeand Variability in Rural West Africa.

[54] Cao C., Chowdhury V.S., Cline M.A., Gilbert E.R. (2021). The Microbiota-Gut-Brain Axis During Heat Stress in Chickens: A Review. Front. Physiol..

[55] Liu G., Zhu H., Ma T., Yan Z., Zhang Y., Geng Y., Zhu Y., Shi Y. (2020). Effect of chronic cyclic heat stress on the intestinal morphology, oxidative status and cecal bacterial communities in broilers. J. Therm. Biol..

[56] Rizzatti G., Lopetuso L.R., Gibiino G., Binda C., Gasbarrini A. (2017). Proteobacteria: A Common Factor in Human Diseases. Biomed. Res. Int..

[57] Tang L.P., Li W.H., Liu Y.L., Lun J.C., He Y.M. (2021). Heat stress aggravates intestinal inflammation through TLR4-NF-kappaB signaling pathway in Ma chickens infected with Escherichia coli O_157_:H_7_. Poult. Sci..

[58] Faivre L. (2018). Safmannan® Helps Counteract *E. coli* Negative Effects in Poultry. Poultry Industry. https://en.engormix.com/poultry-industry/bacterial-diseases-poultry/safmannan-helps-counteract-coli_a42815/.

[59] Posadas G.A., Broadway P.R., Thornton J.A., Carroll J.A., Lawrence A., Corley J.R., Thompson A., Donaldson J.R. (2017). Yeast Pro- and Paraprobiotics Have the Capability to Bind Pathogenic Bacteria Associated with Animal Disease. Transl. Anim. Sci..

[60] Roussel C., Sivignon A., de Vallee A., Garrait G., Denis S., Tsilia V., Ballet N., Vandekerckove P., Van de Wiele T., Barnich N. (2018). Anti-infectious properties of the probiotic *Saccharomyces* cerevisiae CNCM I-3856 on enterotoxigenic *E. coli* (ETEC) strain H10407. Appl. Microbiol. Biotechnol..

[61] Wang W., Li Z., Han Q., Guo Y., Zhang B., D’Inca R. (2016). Dietary live yeast and mannan-oligosaccharide supplementation attenuate intestinal inflammation and barrier dysfunction induced by Escherichia coli in broilers. Br. J. Nutr..

[62] Fathima S., Hakeem W.G.A., Shanmugasundaram R., Selvaraj R.K. (2022). Necrotic Enteritis in Broiler Chickens: A Review on the Pathogen, Pathogenesis, and Prevention. Microorganisms.

[63] Svobodová I., Steinhauserová I., Nebola M. (2007). Incidence of *Clostridium perfringens* in Broiler Chikens in the Czech Republic. Acta Vet. Brno.

[64] Abd El-Hack M.E., El-Saadony M.T., Elbestawy A.R., El-Shall N.A., Saad A.M., Salem H.M., El-Tahan A.M., Khafaga A.F., Taha A.E., AbuQamar S.F. (2022). Necrotic enteritis in broiler chickens: Disease characteristics and prevention using organic antibiotic alternatives—A comprehensive review. Poult. Sci..

[65] Granstad S., Kristoffersen A.B., Benestad S.L., Sjurseth S.K., David B., Sorensen L., Fjermedal A., Edvardsen D.H., Sanson G., Lovland A. (2020). Effect of Feed Additives as Alternatives to In-feed Antimicrobials on Production Performance and Intestinal *Clostridium perfringens* Counts in Broiler Chickens. Animals.

[66] Hofacre C.L., Beacorn T., Collett S., Mathis G. (2003). Using competitive exclusion, mannan-oligosaccharide and other intestinal products to control necrotic enteritis. J. Appl. Poult. Res..

[67] Ao Z., Kocher A., Choct M. (2012). Effects of Dietary Additives and Early Feeding on Performance, Gut Development and Immune Status of Broiler Chickens Challenged with *Clostridium perfringens*. Asian-Australas. J. Anim. Sci..

